# Characterization of the complete chloroplast genome of *Saussurea integrifolia* (Compositae)

**DOI:** 10.1080/23802359.2019.1675547

**Published:** 2019-10-11

**Authors:** Chunlin Chen, Yihua Gong, Guoqian Hao

**Affiliations:** a Key Laboratory for Bio-resource and Eco-environment of Ministry of Education, College of Life Sciences, Sichuan University, Chengdu, Sichuan, PR China;; b Administration Office of Meigu Dafengding National Nature Reserve, Meigu, Sichuan, PR China;; c Biodiversity Institute of Mount Emei, Mount Emei Scenic Area Management Committee, Leshan, China

**Keywords:** *Saussurea integrifolia*, Compositae, chloroplast genome, phylogenetic tree

## Abstract

The complete chloroplast genome sequence of *Saussurea integrifolia*, a flowering plant occurring in Hengduan Mountains with high altitudes, is determined in this study. The plastome is 152,584 bp in length, with one large single-copy region of 83,497 bp, one small single-copy region of 18,646 bp, and two inverted repeat (IR) regions of 25,221 bp. It contains 123 genes, including 86 protein-coding genes, 8 ribosomal RNA, and 36 transfer RNA. Phylogenetic tree shows that this species is a sister to *Arctium lappa*. The published plastome within *Saussurea* provides significant insight for elucidating the phylogenetic relationship of taxa within tribe Compositae.

*Saussurea integrifolia* Hand.- Mazz. is a perennial herbaceous plant of Compositae, and grows in damp shady regions at an altitude from 1600 to 3800 m.s.a.l. *Saussurea*, a highly diversified genus with about 300 species in the Compositae, has been an extremely difficult group in both taxonomical and phylogenetic studies (Wang and Liu 2004; Li et al. 2012). In our study, we characterized the complete chloroplast genome sequence of *S. salicifolia* for further physiological, molecular. and phylogenetical study of this species.

Fresh leaves of *S. integrifolia* were collected from Xiaozhaizigou National Reserve (Mianyang, Sichuan, China; coordinates: 103°45′E, 31°50′N) and dried with silica gel. The voucher specimen was stored in Sichuan University Herbarium with the accstion number of ZL208. Total genomic DNA was extracted with a modified CTAB method (Doyle and Doyle 1987). First, we obtained 10 million high-quality pair-end reads for *S. integrifolia*, and after removing the adapters, the remained reads were used to assemble the complete chloroplast genome by NOVOPlasty (Dierckxsens et al. 2017). The complete chloroplasts genome sequence of *S. polylepis* was used as a reference. Plann v1.1 (Huang and Cronk 2015) and Geneious v11.0.3 (Kearse et al. 2012) were used to annotate the chloroplasts genome and correct the annotation. The complete cp genome has been submitted to GeneBank (accession number MN013403).

The *S. integrifolia* cp genome is 152,584 bp in length, exhibits a typical quadripartite structural organization, consisting of a large single-copy (LSC) region of 83,497 bp, two inverted repeat (IR) regions of 25,221 bp, and a small single-copy (SSC) region of 18,646bp. The cp genome contains 123 complete genes, including 86 protein-coding genes (86 PCGs), 8 ribosomal RNA genes (four rRNAs), and 36 tRNA genes (28 tRNAs). Most genes occur in a single copy, while 18 genes occur in double, including all rRNAs (4.5S, 5S, 16S, and 23S rRNA), 7 tRNAs (trnA-UGC, trnI-CAU, trnI-GAU, trnL-CAA, trnN-GUU, trnR-ACG, and trnV-GAC), and 7 PCGs (rps7, rps12, rpl2, rpl23, ndhB, ycf2 and ycf15). The overall AT content of cp DNA is 62.3%, while the corresponding values of the LSC, SSC, and IR regions are 64.2%, 68.6%, and 56.8%, respectively.

In order to further clarify the phylogenetic position of *S. integrifolia*, plastome of five representative *Saussurea* species were obtained from NCBI to construct the plastome phylogeny, with *Atractylodes chinensis* as an outgroup. All the sequences were aligned using MAFFT v.7.313 (Katoh and Standley 2013) and maximum likelihood phylogenetic analyses were conducted using RAxML v.8.2.11 (Stamatakis 2014). The phylogenetic tree shows that *S. integrifolia* clustered together with *Arctium lappa*. The *Cynara humilis* clustered together with *Silybum marianum* and *Cirsium vulgare*, and then formed one clade within the Compositae (Figure 1).

**Figure 1.**
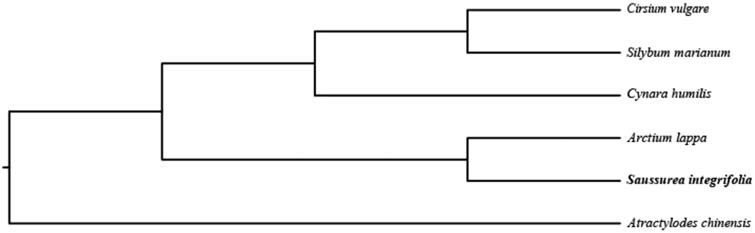
Phylogenetic relationships of Musaceae species using whole chloroplast genome. GenBank accession numbers: *Cirsium vulgare* (NC_036967), *Arctium lappa* (MH375874), *Atractylodes chinensis* (NC_037484), *Cynara humilis* (KP299292), *Silybum marianum* (NC_028027).
